# Detection of *Chlamydia trachomatis ompA* DNA in urine by loop-mediated isothermal amplification (LAMP) assay

**DOI:** 10.1515/almed-2024-0117

**Published:** 2025-02-05

**Authors:** Hemali Attanayake, Charitha Goonasekara, Nalaka Abeygunasekera, Jayanthi Elvitigala, Kamani Mangalika Gunasekera

**Affiliations:** National STD AIDS Control Program, Colombo, Sri Lanka; Department of Pre Clinical Sciences, Faculty of Medicine, General Sir John Kotelawala Defence University, Ratmalana, Sri Lanka; STD Clinic, Colombo South Teaching Hospital, Kalubowila, Sri Lanka; Department of Microbiology, Faculty of Medical Sciences, University of Sri Jayewardenepura, Nugegoda, Sri Lanka

**Keywords:** *Chlamydia trachomatis*, crude extract, loop mediated isothermal assay (LAMP), urine

## Abstract

**Objectives:**

Efficient real-time PCR kits are commercially available for the detection of *Chlamydia trachomatis* (CT) genetic material, but at a price. As a result, cost-effective, sensitive and specific CT diagnostic tests are essential for resource limited countries. This study aims to describe the optimization of a loop mediated isothermal amplification (LAMP) assay for the detection of CT *ompA* DNA in urine.

**Methods:**

Cost-saving modifications included using *Bsm* polymerase and a nucleic acid gel stain. Crude DNA extraction (method-1) involved centrifuging urine at 14,000 g for 30 min, heating the deposit at 95 °C for 5 min, and centrifuging again at 17,000 *g* for 1 min. To boost sensitivity, urinary inhibitors were diluted with phosphate-buffered saline washes and a larger urine volume was used (method-2). The LAMP-SYBR GOLD assay was incubated at 56 °C for 60 min, with nucleic acid gel stain color changes observed under UV light. Urine from 326 sexually transmitted diseases clinic attendees was tested with both LAMP-SYBR GOLD and real-time PCR, comparing sensitivity and specificity.

**Results:**

Analytical sensitivity of the LAMP-SYBR GOLD assay was 0.8 copies per reaction volume. Compared to real-time PCR, LAMP-SYBR GOLD assay had sensitivity, specificity, positive predictive and negative predictive values of 71.4 , 99.7, 96.2, 96.7 % respectively. Five of the seven false negative results obtained with method-1 were re-tested using method-2, providing in all the cases the expected positive results.

**Conclusions:**

The LAMP-SYBR GOLD assay showed a sensitivity of 71 % and a high specificity for detecting CT in urine with extraction method-1. The use of method-2 could increase this sensitivity, likely due to the removal of urine inhibitors.

## Introduction

*Chlamydia trachomatis* (CT) is the most common aetiological agent of non-gonococcal urethritis with a worldwide prevalence of 2.9 % [1]. Genito-urinary infections with CT are mostly asymptomatic, leading to complications such as pelvic inflammatory disease, ectopic pregnancy and tubal factor infertility in women and epididymitis and proctitis in men. Both symptomatically and asymptomatically infected individuals transmit infections to sexual partners [1]. In 2020, approximately 129 million new cases of genital CT infections were reported globally [2]. In Sri Lanka, the proportion of CT in women visiting the clinics of National STD and AIDS Control Program (NASCP) increased from 8.3 % in 2012 [3] to 17.1 % in 2015 [4].

Nucleic acid amplification tests (NAATs) are the most sensitive and specific for CT detection and is considered the gold standard test [5]. Although Food and Drug Administration approved commercial NAATs are available for CT diagnosis [5], these are prohibitively expensive for a resource limited country like Sri Lanka. NSACP of Sri Lanka is responsible for coordinating the national response to HIV and sexually transmitted infections in collaboration with many national and international stakeholders. Due to the high cost, real-time PCR tests are performed only on symptomatic patients and asymptomatic high risk individuals. A cost-effective, sensitive and specific test for diagnosis of CT infections, is the need of the hour for Sri Lanka. Loop mediated isothermal amplification (LAMP) is a novel NAAT that is highly sensitive and specific.

Choopara et al. (2017) published a LAMP assay for detecting CT outer membrane protein A (*ompA*) DNA in endocervical swabs which was 91 % sensitive and 95 % specific [6]. The circular genome of CT is a single chromosome containing the *ompA* gene which is 1.2 kb in length. This encodes for the major outer membrane proteins which are used for categorizing CT into 19 genovars [7]. The LAMP primers of Choopara et al. (2017) were designed for the detection of the *ompA* gene, targeting six independent conserved regions [6]. Urine specimens are easier to collect than endocervical swabs and can be used for both sexes. Our objective was to optimize a LAMP method using previously designed primers [6] to detect CT *ompA* DNA in urine.

## Materials and methods

### Collection of specimens

First void urine was collected from male and female patients who attended the clinics of NSACP (n=126) and Colombo South Teaching Hospital, Kalubowila, Sri Lanka (n=200), from 19 March 2018 to 28 October 2019 (total n=326). Criteria for recruitment were: age ≥18 years, symptoms suggestive of urogenital infection (i.e. abnormal urethral/cervical/vaginal discharge, increased white cells in discharge, co-infection with *Neisseria gonorrhoeae*) and asymptomatic patients with risk factors (such as multiple partners, men who have sex with men, male and female sex workers, the clients of sex workers and contacts of patients with cervicitis or urethritis). Patients who had passed urine less than 2 h before recruitment and who had taken antibiotics during the previous three weeks were excluded. For a CT prevalence rate of 13.9 % symptomatic and 9.2 % asymptomatic patients [4], the calculated sample size was 184 symptomatic and 129 asymptomatic patients, with a margin of error of 0.05 and a 95 % confidence interval (statistical formula http://www.calculator.net/sample-size-calculator.html). Prior to physical examination of patients, 30 mL of first void urine was collected and stored at 2–8 °C, for not more than seven days before processing. Aliquots of fresh voided urine were stored at −80 °C for repeat testing during optimization.

### Positive and negative control urine

The positive control urine sample was a urine that tested positive, by both the nested PCR [8] and the Artus^®^ *C. trachomatis* Plus RG Real time PCR (Qiagen, Germany); the negative control urine sample was a urine that tested negative by both these tests. The positive/negative control urine samples were aliquoted and stored at −20 °C and were used for all experiments.

### Crude DNA extraction for LAMP-SYBR GOLD assay (method-1)

Crude DNA extraction for LAMP-SYBR GOLD assay was as described by Choopara et al. with a few modifications (method-1) [6]. Urine (1.5 mL) was centrifuged at 14,000 *g* for 30 min. Supernatant was discarded and 40 µL of urine deposit was heated with 20 µL of 10 mM Tris-HCl and 1 mM EDTA buffer (pH 7.5) at 95 °C for 5 min using a heating block [6]. It was cooled on ice until the liquid cleared on top, centrifuged at 17,000 *g* for 1 min and the supernatant was used for LAMP-SYBR GOLD assay. Crude extracts were prepared on the same day as the assay.

### Crude DNA extraction for LAMP-SYBR GOLD assay (method-2)

To increase the sensitivity of the LAMP-SYBR GOLD further, modifications were done to method-1. Stored aliquots of urine (1.5 mL) were thawed rapidly in a water bath at 37 °C, followed by centrifugation at 14,000 *g* for 30 min at room temperature. The supernatant was discarded and 200 µL of phosphate buffered saline (PBS; pH 7.4) was added to dilute the urinary inhibitors, and centrifuged at 14,000 *g* for 30 min at room temperature. Following removal of the supernatant the pellet was dissolved in 200 µL of PBS and heated at 95 °C for 5 min with 100 µL of Tris-EDTA (pH 7.4) buffer. Tubes were cooled on ice and centrifuged at 17,000 *g* for 1 min. The supernatant was then collected for LAMP-SYBR GOLD assay analysis.

### Extraction of DNA from urine by QIAamp viral RNA kits

This kit is recommended for the use of DNA extraction from bacteria in urine [9]. Extraction was performed according to the manufacturer’s instructions for the spin protocol. Hundred and 40 μL of urine were lysed and added to the spin columns with buffer and centrifuged. DNA was eluted in the given elution buffer. Aliquots of DNA were stored at −20 °C for repeat assays.

### Real-time PCR

DNA templates were prepared from patient urine using the QIAmp viral RNA mini kit (Qiagen, Germany). The Artus^®^ *C. trachomatis* Plus RG Real time PCR (Qiagen, Germany) was performed according to the manufacturer’s instructions with the provided internal control. Real-time PCR results were used as the reference for determining the sensitivity and specificity of the LAMP-SYBR GOLD assay.

### Nested PCR

A nested PCR described by Somani et al. (2000) was used for confirming the real time PCR results of the positive and negative controls [8]. DNA extracted with QIAamp Viral RNA Mini kit was used and the nested PCR was performed as described by Somani et al. (2000) targeting the CT *omp A* gene [8].

### LAMP-Sybr Gold assay

Positive and negative controls were used for optimizing the LAMP-SYBR GOLD assay. A set of six published primers [6], FIP, BIP, F3, B3, FLP, BLP (Integrated DNA Technologies, USA), targeting the *ompA* gene of CT, was utilized for the LAMP-SYBR GOLD assay [6]. Optimal LAMP-SYBR GOLD assay conditions were determined for the following components; Betaine (0.5 M, 0.8 M), MgSO_4_ (6–10 mM), *Bsm* DNA polymerase (4U, 6U, 8U), template volume (5–9 µL), incubation temperature (55–60 °C) and reaction time (40 and 60 min), using gel electrophoresis for detection.

The optimized LAMP-SYBR GOLD assay conditions for a 25 µL LAMP reaction were, 7 µL crude extract (method-1) in 1X buffer (20 mM Tris-HCl [pH 8.8], 10 mM KCl, 10 mM (NH_4_)_2_SO_4_, 2 mM MgSO_4_, 0.1 % (v/v) Tween 20), 1.6 µM FIP and BIP primers, 0.2 µM F3 and B3 primers, 1.4 µM FLP and BLP primers [6], 1.4 mM deoxynucleoside triphosphate (Qiagen, Germany) each, 0.8 M Betaine (Sigma Aldrich, USA), 6 mM MgSO_4_, 8U *Bsm* polymerase large fragment (Thermo Scientific, USA). Before adding the crude extract, 12.5 µL of liquid paraffin was layered on top of the master mix and the template was deposited below this layer. One microliter of 10X Sybr™ Gold nucleic acid gel stain (Invitrogen, USA; Cat. No. S11494) was suspended from the inside of the lid of the reaction tube. It was incubated at 56 °C for 60 min and the reaction was stopped by heating to 80 °C for 2 min. At the end of incubation, the Sybr™ Gold stain (Invitrogen, USA; Cat. No. S11494) was incorporated in the reaction mix by vortexing. Positive reactions were detected by bright yellow flourescence under 320 nm UV illumination.

### Analytical sensitivity and specificity

CT DNA extracted with QIAmp viral RNA mini kit (Qiagen, Germany), was quantitated using NanoDrop^®^ Spectrophotometer. Analytical sensitivity of the optimized LAMP-Sybr Gold assay was determined using negative control urine, spiked with 10-fold serial dilutions of quantitated CT DNA (7.49 ng–7.49 × 10^−4^ fg; 8.06 × 10^9^–8.06 × 10^−1^ copies). The LAMP-SYBR GOLD assay product was visualized using 320 nm UV light and 1.5 % gel electrophoresis stained with Sybr™ Gold stain (Invitrogen, USA; Cat. No. S11494). Specificity of primers was confirmed by performing LAMP-Sybr Gold assay with negative control urine spiked with DNA from herpes simplex virus type 2, *N. gonorrhoeae*, *Trichomonas vaginalis, Candida albicans* (obtained from NSACP, Sri Lanka) and *Mycoplasma genitalium* DNA.

### Diagnostic sensitivity and specificity

LAMP-Sybr Gold assay with crude extract (method-1) was evaluated using 326 first void urine samples collected from 189 symptomatic patients and 137 asymptomatic high risk patients. The investigator performing the LAMP-SYBR GOLD assay was blinded to the results of real time PCR assay which was the reference test. Results were recorded by visualizing the color change with the UV lamp. Discordant results in LAMP-SYBR GOLD assay were repeated.

## Results

### Optimization of LAMP-SYBR GOLD assay

Different methods of DNA extraction were tried during initial optimization. DNA from sodium hydroxide extraction gave a low limit of detection and DNA extracted by heating and alcohol precipitation had poor purity (data not shown). Since the crude extraction method described by Choopara et al. gave the best results compared to above methods we tried, extraction method-1 was used for optimization of the LAMP-SYBR GOLD assay [6]. Optimization of MgSO_4_ concentration (see Supplementary Material, Figure 1) revealed that the most distinct ladder pattern occurred at a concentration of 8 mM. The optimization of incubation temperature (Supplementary Figure 2A) indicated that 56 °C was the optimal temperature, while in Supplementary Figure 2B, the clearest band was observed with 7 µL of template after 60 min. The estimation of the limit of detection (LOD), using different concentrations of Bsm polymerase (Supplementary Material, Figure 3) and varying template volumes (Supplementary Material, Figure 4), showed that the optimal conditions were 8U of Bsm polymerase and 7 µL of template. Each optimization test was conducted in triplicate. Visual colour indicators, hydroxy naphthol blue and ethidium bromide were evaluated with this LAMP assay, but the color change at end points was difficult to discern. Therefore, Sybr™ Gold nucleic acid gel stain (Invitrogen, USA; Cat. No. S11494) was used with UV light. Sybr™ Gold stain emits a yellow fluorescence under UV light, when bound to DNA (Supplementary Material, Figure 5).

### Limit of detection (LOD)

When crude DNA extraction from urine was performed exactly as described by Choopara et al. the LOD was 74.9 fg (8.06 × 10^4^ copies) by gel electrophoresis [6]. Touchdown LAMP only increased the LOD 10-fold (data not shown). In order to increase the LOD further, urine samples were centrifuged before and after heating (method-1) and Sybr™ Gold nucleic acid gel stain (Invitrogen, USA; Cat. No. S11494) was added as the visual indicator. As a result of these modifications to the method described by Choopara et al., the LOD improved from the initial 74.9 fg to 7.49 × 10^−4^ fg, or approximately 0.8 copies, when visualized under UV light. The LOD was the same by UV light visualization as well as gel electrophoresis. Therefore, when testing the clinical samples, UV light visualization was used for detecting the amplicon.

### Diagnostic sensitivity and specificity

When 326 urine specimens were screened with LAMP-SYBR GOLD assay and crude extraction (method-1), one false positive and seven false negatives were detected; real time PCR was the reference test (Table 1). This assay had 71.4 % (20/28) sensitivity, 99.7 % (297/298) specificity, 96.2 % positive predictive value, 96.7 % negative predictive and 97.2 % accuracy (Table 1).

**Table 1:** Results of 326 urine specimens screened by LAMP-Sybr Gold with crude extract (method-1) and real time PCR.

	Real time PCR positive	Real time PCR negative	Total
LAMP with crude extract (method-1) positive	20	1	21
LAMP with crude extract (method-1) negative	8	297	305
Total	28	298	326

Six of the seven false negatives and the one false positive were seen in symptomatic patients. In both symptomatic and asymptomatic patients, the sensitivity and specificity of LAMP Sybr-Gold assay were similar (Table 2). The LAMP-SYBR GOLD assay showed no cross-reactivity with other genital pathogens, including herpes simplex virus type 2, *N. gonorrhoeae, M. genitalium, T. vaginalis*, and *C. albicans*, as indicated by the lack of a ladder patterns in the gel (see Supplementary Material, Figure 6). Five of the seven false negative specimens were re-tested with crude extraction method-2. All five samples became weakly positive by UV light, and was confirmed by the ladder pattern seen in gel electrophoresis (Supplementary Material, Figure 7).

**Table 2:** Urine specimens of symptomatic and asymptomatic patients tested with real-time PCR and LAMP-SYBR GOLD with crude extraction method-1.

	Real-time PCR		Total
	Positive	Negative	
**Symptomatic patients** (n*=*189)			

LAMP + crude extract (method-1) positive	17	1	18
LAMP + crude extract (method-1) negative	7	164	171
Total	24	165	189
Sensitivity 70.8 %			
Specificity 99.4 %			

**Asymptomatic patients** (n=137)			

LAMP + crude extract (method-1) positive	3	0	3
LAMP + crude extract (method-1) negative	1	133	134
Total	4	133	137
Sensitivity 75 %			
Specificity 100 %			

## Discussion

Choopara et al. (2017) developed a highly sensitive (91 %) and specific (95 %) LAMP assay for detecting CT in endocervical swabs [6]. As urine is a less invasive and easy to collect specimen which can be used in both sexes, we made modifications to the method by Choopara et al. (2017) that would enable detection of CT in urine. Utilizing previously published primers [6], we developed a LAMP-SYBR GOLD assay for urine. Our LAMP-SYBR GOLD had 71.4 % sensitivity, 99.7 % specificity and a LOD of 0.8 copies of CT *ompA* DNA per reaction, with Sybr™ Gold stain as the visual indicator (Supplementary Material, Figure 6).

One of the modifications made to the protocol by Choopara et al., was the use of *Bsm* polymerase [6]. *Bsm* polymerase is one third the price of *Bst* polymerase and more widely available in Sri Lanka. With the intention of reducing the cost further, the loop primers were not HPLC purified. These modifications may have led to the 15-min extension of the incubation period. When we applied the protocol by Choopara et al. (2017), with the above modifications to urine, the LOD was 8.06 × 10^4^ copies of CT *ompA* DNA compared to the LOD of 1.8 × 10^2^ copies which was achieved by the original protocol for endocervical specimens [6]. Touchdown LAMP was used in an attempt to increase the analytical sensitivity but it produced only a 10-fold increase (data not shown). Some modifications were done to the method of extraction described by Choopara et al. (method-1): (1) urine was centrifuged at 14,000 g for 30 min and the deposit was collected for heat extraction, (2) following heat extraction, it was centrifuged at 17,000 g for 1 min and the supernatant was collected for LAMP-SYBR GOLD analysis. This modification to the crude DNA extraction, increased the LOD significantly. Sybr™ Gold nucleic acid gel stain (Invitrogen, USA; Cat. No. S11494) is 25–100 times more sensitive than ethidium bromide [10] and can be used at a high dilution. Modification of the extraction method, high sensitivity of Sybr™ Gold stain (Invitrogen, USA; Cat. No. S11494), and large (7 µL) template volume, contributed to the increase in LOD to 8.06 × 10^−1^ (0.8) copies. DNA contamination was prevented by layering paraffin wax over the master mix and depositing the template below the layer of wax.

Urinary inhibitory factors in the crude genomic DNA template may have been one of the reasons for the low clinical sensitivity, despite the LOD of 0.8 copies of CT *ompA* DNA per reaction. Secondly, the amount of genomic DNA released by heating 40 µL of urinary deposit may have been inadequate. Optimizing the volume of urinary deposit used for heat extraction is necessary. Urinary inhibitors are responsible for the reduced efficiency of LAMP assays in urine. Edward et al. (2014) determined that LAMP reactions could withstand higher levels of urea than those found in human urine and Jevtuševskaja et al. (2016) established that bovine serum albumin, Mg^2+^ and urea at physiological amounts do not affect the LAMP process [11], [12]. In our analytical sensitivity test, CT negative urine was spiked with purified CT DNA. As such, the low clinical sensitivity seen in our LAMP-SYBR GOLD assay is unlikely to be due to urea.

In crude extraction method-2, in order to remove possible urinary inhibitors, the urinary deposit was centrifuged with PBS (washing step). This method was applied to five patient samples which were positive by real time PCR, but negative with crude extraction method-1. Weakly positive results in all five patient samples, suggest that removal of urinary inhibitors by method-2 could improve the sensitivity of the LAMP-SYBR GOLD assay (Supplementary Material, Figure 7). Following optimization of the amount of urinary deposit, all patient samples need to be retested with extraction method-2, to determine the true clinical sensitivity of this LAMP-SYBR GOLD assay. The challenge of developing diagnostic assays for resource limited countries is balancing the cost against the efficacy of the test. Although commercial extraction kits improve the sensitivity of LAMP assays, the very high cost makes it unsuitable for poor countries. As proven by several studies crude extraction of DNA by heating is rapid, inexpensive and adequate for CT LAMP assays [6], [12].

Since the introduction of the LAMP method by Notomi et al. (2000), there have been very few CT LAMP assays developed, and these have been for endocervical specimens [6], [8], [13], [14], [15], [16]. The only CT LAMP assay developed for urine by Jevtuševskaja et al. (2016) required pre-treatment with an antimicrobial peptide lysis mix and was 73 % sensitive and 100 % specific with a LOD of 25 plasmid copies/reaction [12]. The commercial availability of highly effective CT real time PCR kits, has led to the neglect of attempts to develop less expensive diagnostic methods for resource limited countries. Our LAMP-SYBR GOLD assay for urine was sensitive and highly specific for the detection of CT *ompA* DNA in urine with Sybr™ Gold stain as a visual indicator. This method is affordable (cost of reagents for a single specimen was approximately one US Dollar), simple to perform and better suited for resource limited countries. The entire process required a little more than one and a half hours. Our findings demonstrate that CT LAMP-SYBR GOLD assay on urine, is a cost-effective method which could be further improved. We optimized and clinically validated the LAMP-SYBR GOLD assay using extraction method-1. However, we could not achieve the same for extraction method-2. While method-2 shows promise, its effectiveness still requires validation through independent studies. For countries that cannot afford the high cost of highly efficient real-time PCR, this LAMP-SYBR GOLD method would be a less expensive alternative for CT diagnosis.

## Supplementary Material

Supplementary Material
