# Supplementary material for: Detection of Chlamydia trachomatis ompA DNA in urine by loop-mediated isothermal amplification (LAMP) assay
Source: Adv Lab Med. 2025 Feb 5;6(3):314–9. doi: 10.1515/almed-2024-0117 (PMC12446907; doi:10.1515/almed-2024-0117)
Supplement: Supplementary file 1 — Supplementary Material [file j_almed-2024-0117_suppl_001.docx]

**
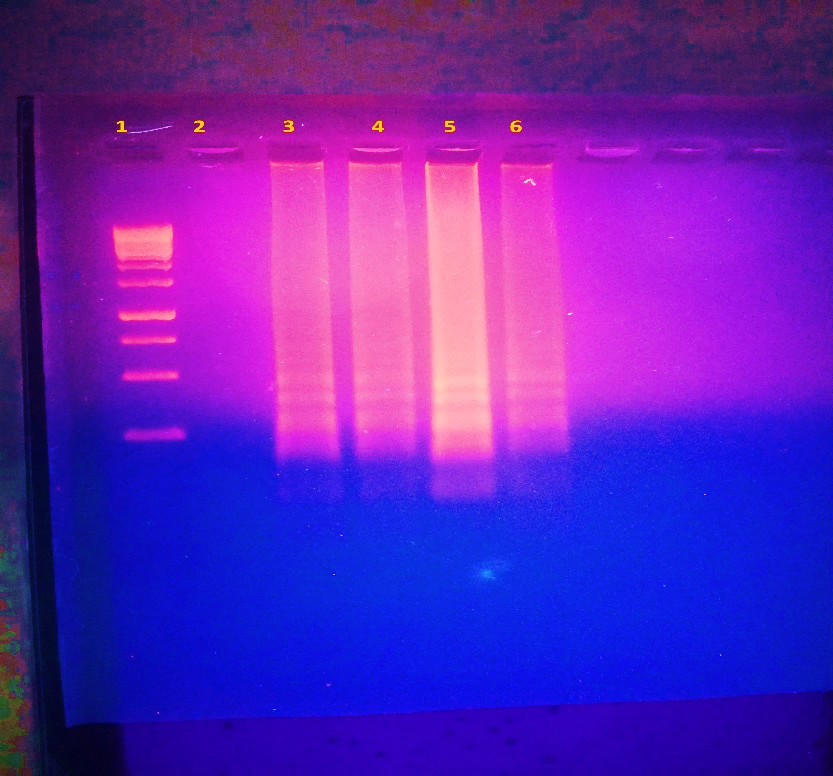
**

bp

10000

8000

6000

5000

4000

3000

2500

2000

1000

**Supplementary material - Figure 1.** Optimization of MgSO_4_ concentration using gel electrophoresis as the detection method. Lane 1, 1 kb DNA ladder; lane 2, empty; lane 3, 6mM MgSO_4_; lane 4, 7mM MgSO_4_; lane 5, 8mM MgSO_4_ (**optimum**); lane 6, 10mM MgSO_4_; lane 7, negative control with 8mM MgSO_4_.


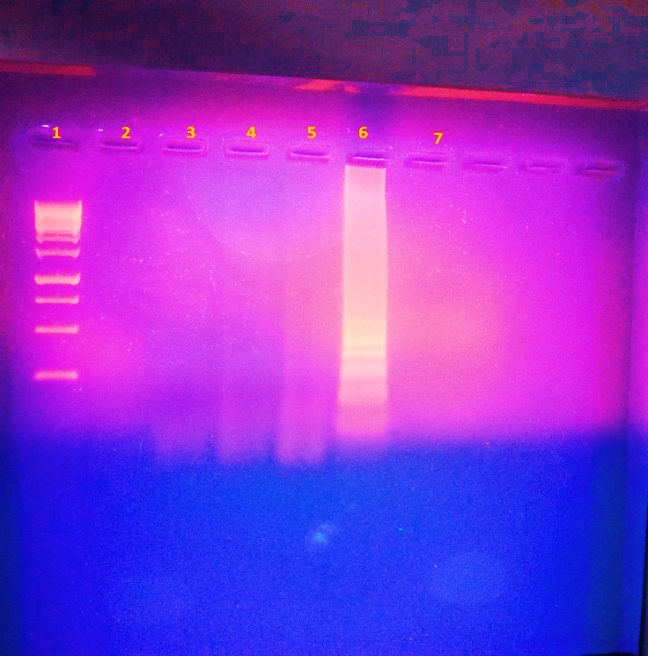
**
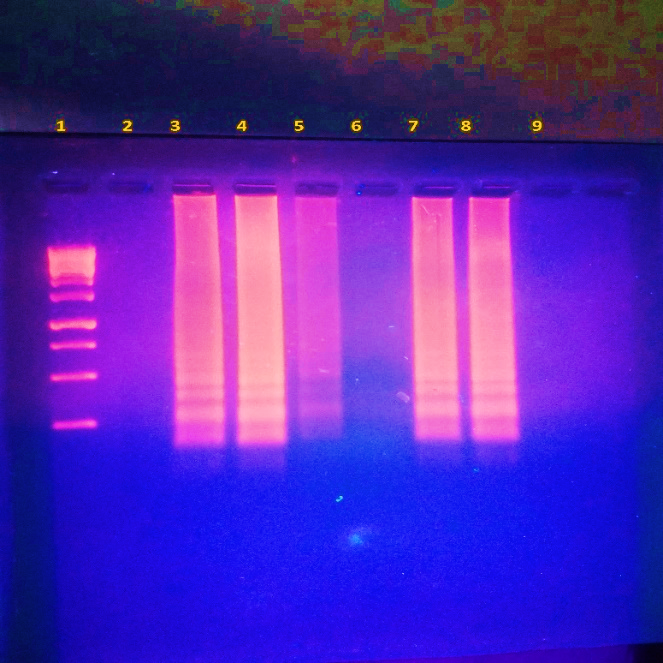
**

B

A

10000

6000

5000

4000

3000

2500

1000

**Supplementary material - Figure 2.** (A) Optimization of LAMP for incubation temperature (55^o^C-60^o^C). Lane 1, 1 kb DNA ladder; lane 2, empty; lane 3, 55^o^C; lane 4, 56^o^C (optimum); lane 5, 57^o^C; lane 6, 58^o^C; lane 7, 59^o^C; lane 8, 60^o^C; lane 9, negative control at 56^o^C. (B) Optimization of duration of incubation (40 & 60 minutes) at 56^o^C with 5-7 µl of template at different concentrations. Lane 1, 1kb DNA ladder; lane 2, empty; lane 3, 5µl of 10^-2^ dilution of positive control (PC) incubated for 40 minutes; lane 4, 6µl of 10^-2^ dilution of PC incubated for 40 minutes; lane 5, 7µl of 10^-2^ dilution of PC incubated for 40 minutes; lane 6, 7µl of 10^-3^ dilution of PC incubated for 60 minutes (**optimum**); lane 7, 7µl of negative control incubated for 60 minutes.

**
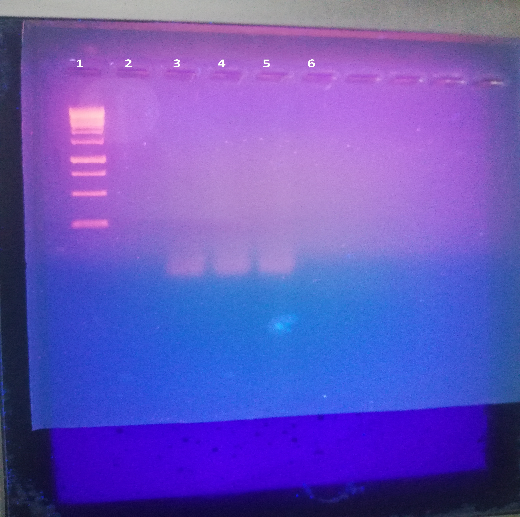

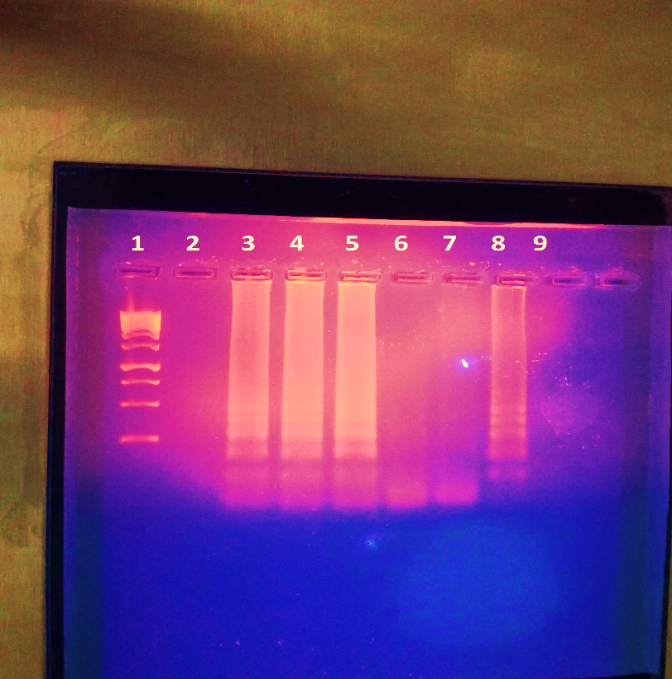
**

B

A

bp

10000

6000

5000

4000

3000

2500

**Supplementary material - Figure 3**. (A) Optimization of LAMP for *Bsm* polymerase (4-6U). Lane 1, 1kb DNA ladder; lane 2, empty; lanes 3-5, 4U, 6U, 8U of enzyme with positive control diluted 10^-1^; lane 6-8, 4U, 6U, 8U (**optimum**) of enzyme with positive control diluted 10^-2^; lane 9, negative control with 8U of enzyme. (B) Lane 1, 1kb DNA ladder; lane 2, empty; lanes 3-5, 4U, 6U, 8U of enzyme with positive control diluted 10^-3^; lane 9, negative control with 8U of enzyme.


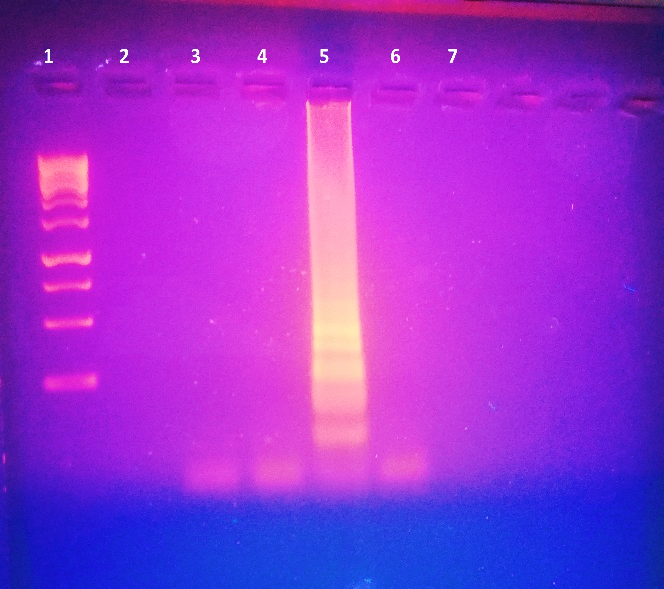


bp

10000

6000

5000

4000

3000

2500

2000

B


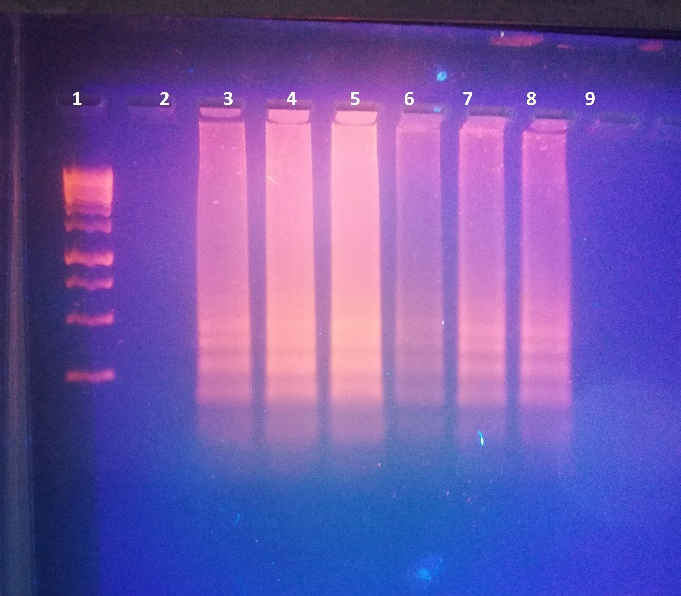
**Supplementary material - Figure 4**. (A) Optimization of LAMP for volume of template. Lane 1, 1kb DNA ladder; lane 2, empty; lanes 3-5, 5µl, 6µl, 7 µl of 10^-1^ dilutions of positive control; lanes 6-8, 5µl, 6µl, 7 µl of 10^-2^ dilutions of positive control; lane 9, 5µl of negative control undiluted. (B) Lane 1, 1 kb DNA ladder; lane 2, empty; lanes 3-5, 5µl, 6µl, 7µl **(optimum)** of 10^-3^ dilutions of positive control; lane 6, 7µl of 10^-4^ dilutions of positive control; lane 7, 7µl of negative control undiluted.

A

A

BB


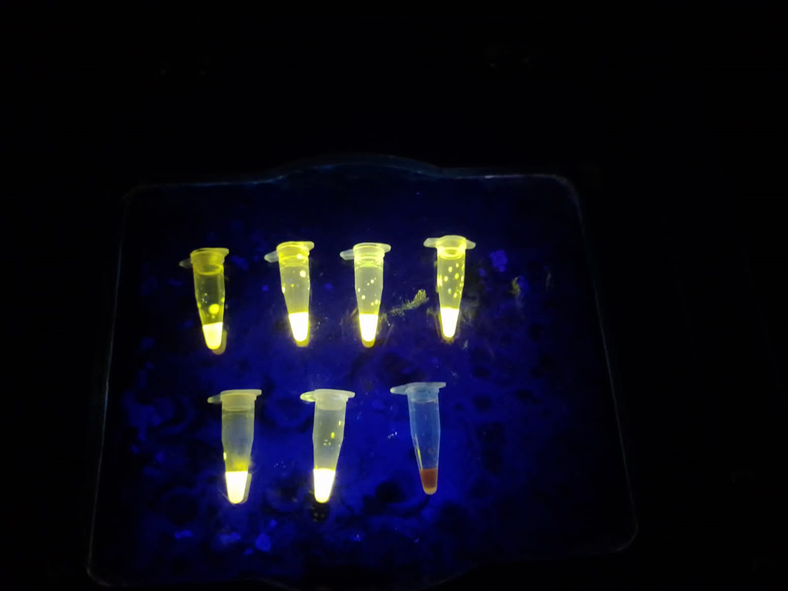


**Supplementary material - Figure 5**. Positive (A) and negative (B) controls of LAMP with Sybr™ Gold nucleic acid stain as observed under UV light.


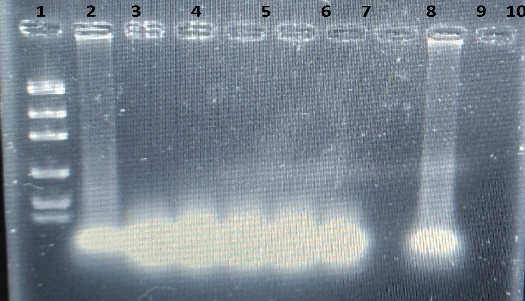


bp

10000

5000

4000

3000

2500

2000

10^-10^  10^-11^ 10^-12^

**Supplementary material - Figure 6**. Specificity of primers were confirmed by urine spiked with DNA from herpes simplex virus type 2 (lane 3), *Neisseria gonorrhoeae* (lane 4), *Mycoplasma genitalium* (lane 5), *Trichomonas vaginalis* (lane 6), *Candida albicans* (lane 7), empty (lane 8), positive control (lane 2 & 9).


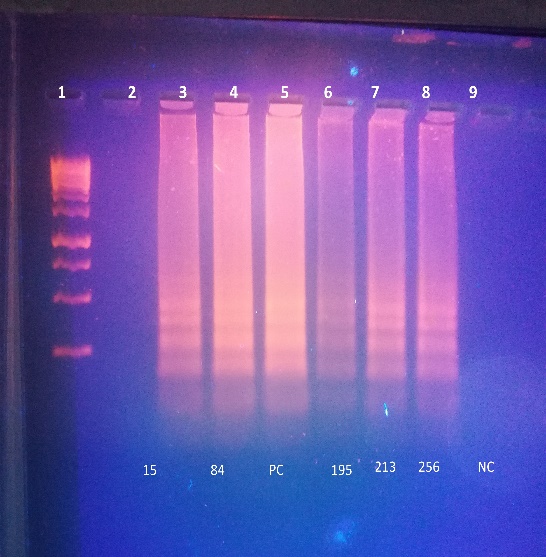


bp

10000

6000

5000

4000

3000

2500

2000

**Supplementary material - Figure 7**. Five false negative specimens (specimen nos. 15, 84, 195, 213 and 256) became weakly positive (ladder pattern) when crude extraction method-2 was applied. Increasing the of volume of urine for extraction and dilution of urinary inhibitors were additional steps used in method-2.
